# Supplementary material for: Zero fruits/vegetables consumption and associated factors among Children aged 6–23 months in Ethiopia: Mixed effect logistic regression analysis
Source: PLoS One. 2023 Jul 14;18(7):e0288732. doi: 10.1371/journal.pone.0288732 (PMC10348598; doi:10.1371/journal.pone.0288732)
Supplement: Attachment — Submitted filename: Magnitude of zero fruits.docx [file pone.0288732.s002.docx]

**Magnitude of zero fruits/Vegetable consumption and associated factors among children aged 6-23 months in Ethiopia**

Summary: dig out such developing countries problem is important but showing the magnitude at local level is more significant, because the data obtained from this research finding is almost defined in the DHS report also.

**Background:** line 63: better to rephrase it.

**Methodology:** Line 146-149: you mentioned a contradictory idea with your justification. Amendment on your justification is required. If it is so your study doesn’t show any new findings than the previous studies you cited. Do you think wealth index is an individual level variable? Did you generate WI again or you took from the report of EMDHS? When you classify poor, middle or rich, the minimum required number??? When we say there is media exposure, better to operationalized???

Line 161: what is your weighting number???

Line165: Have checked it and violated the assumptions or based on your statistical knowledge?

Line 172-73: the ultimate use of LLR, deviance or AIC is similar. Why not you used either of one???

Line 193: large central region??? Better to operationalize.

**Result, discussion**

**Line 198**: Most of sample were from poor house household….. Will biased your findings. Needs management. Narration and table similar…..

**Line 206:** Is the 95% confidence interval wide or narrow? Why?

**Line 215-table: 1**-have checked chi-square assumptions? And if the difference in the geopolitical regions were significant, what did you think about the regression you did?

**Line 228-229**: Does the interpretation give a logical sense???

**Table 2:** if you obtain similar values for them, and between them, which is better for multilevel model comparison if any?

**Table 3:** Your statistical significant variables are mostly sociodemographic and economic factors. What do you feel about these and its implications?

**Line 281-285:** Dear authors, are you sure your findings are consistent with the cited reference findings??? If it is so, what is the criteria to say consistent, inline, higher, lower, opposite etc.?

**Line 289-291**: if the participants were different, can we use them for comparison?

**Line 296:** most of your participants are from poor households…… it is difficult to assure a true statistical significance……… if the sample size for all categories is high, it might not be significant. Do you have any method you used for statistical significant assurance???

Line 329: Your interpretation is inappropriate, the religion you mentioned does not recommend fasting for populations like your participants. Needs revision or better justification.
