# Supplementary material for: Zero fruits/vegetables consumption and associated factors among Children aged 6–23 months in Ethiopia: Mixed effect logistic regression analysis
Source: PLoS One. 2023 Jul 14;18(7):e0288732. doi: 10.1371/journal.pone.0288732 (PMC10348598; doi:10.1371/journal.pone.0288732)
Supplement: Attachment — Submitted filename: Response to Reviewers.docx [file pone.0288732.s003.docx]

Manuscript PONE-D-23-02355

PLOS ONE Response to Reviewers

Dear Dr. Melaku Kindie Yenit
Academic Editor
PLOS ONE

Thank you for giving us the opportunity to submit a revised draft of the manuscript “Magnitude of Zero Vegetable / Fruit Consumption and Associated Factors among Children age 6-23 months in Ethiopia: Mixed effect logistic regression analysis” for publication in the PLOS ONE journal. We appreciate the time and effort that you and the reviewers dedicated to providing feedback on our manuscript and are grateful for the insightful comments on and valuable inputs you have provided, which significantly improved the manuscript. We have incorporated most of the suggestions made by the reviewers. Those changes are highlighted within the revised manuscript with track changes. Please see below, in the table, for a point-by-point response to the reviewers’ comments and concerns. **All line numbers refer to the revised manuscript file without tracked changes.**

| **Academic Editor comment** | **Response** |
| --- | --- |
| Dear Authors,  Thank you again for your manuscript submission.  Your manuscript has now been reviewed by experts in the field. Please have a look in the attachment section and act accordingly. | We are glade to submit our work to PLOS ONE.  Thank you! All the concerns raised by the experts are responded point by point in the following tables. |

| **Reviewer#1 comments** | **Responses** |
| --- | --- |
| Summary: I hereby testify that I am very much delighted to read and contribute my part for the betterment of your manuscript. The issue you raised is a new indicator for evaluating the under-two children's minimum dietary diversity status in the 2021 WHO/UNICEF IYCF Guideline. I found your work is scientifically relevant and will yield a baseline evidence for Ethiopia's IYCF status pertaining to vegetables/fruits consumption in under-two years children. Please be advised that you need to incorporate or take in to consideration of my issues or concerns while you work on the manuscript revision. | Thank you! And all the concerns you raised to improve our manuscripts is incorporated below. |
| 1) Title: Have you assessed vegetable only or fruits only or both? What if some eats vegetable but not fruits and vice versa? | We assessed both fruits and vegetable consumption. If the child eats none of the food groups, he/she is considered as the one who consumes zero fruits/vegetables |
| 2) Please correct your Affiliation as: Amhara, not Ahara | Thank you for pointing this out. This has been corrected. |
| 3) How do you generate keywords? The keywords you stated are already presented in the title; what is the need to repeat them? | Thank you for pointing this out. We generated the keywords with the goal of accurately reflecting the content of the manuscript and help other researchers find our work. To avoid the repetition, we changed some of them, kindly look at line number 49. |
| 4) Please write as 'Methods' or 'Results'; not 'Method' or 'Result' in all sections of the manuscript | We agree with this and have revised accordingly. |
| 5) Where are the multi-level findings in the abstract section of the results? | Thank you for pointing this out. We have incorporated the multi-level findings in the abstract section kindly find it in line number 31-42 |
| 6) Conclusion for the first objective is confusing; not focused | Agree. We have modified to make it focused. Kindly look at line number 44 |
| 7) Background: 1) Not focused to the problem under study; 2) Existing gaps weren't well stated; 3) There is no information about existing factors so far | 1)The indicator is relatively new, and to the best of the authors’ knowledge there is paucity of published articles which deal directly with zero fruits/vegetables consumption.  2)We are grateful for your concern here. We have revised to show the existing gaps better. Kindly look through line number 96 to 105  3) Thank you again for pointing this out. Modified accordingly to show existing factors. Kindly look at line number 92 to 95 |
| 8) Methods: 1) You stated 9 regions, but Ethiopia has 11 regions, currently; you need to specify it; 2) P-value < or = 0.25 in the bivariable analysis were entered in to the multivariable analysis: why you do this since you have small number of variables? | 1)Thank you for pointing this out. This has been revised, and incorporated at line number 117-118  2)Thank you for this suggestion. It would have been better to enter all variables to the multivariable analysis. However, in the case of our study, at the beginning we had nineteen variables and we believed that the number of variables were not small. Also, in order not to lose more variables we used p-value of 0.25 instead of 0.2 |
| 9) Results: 1) What is your focus: 'Prevalence' or 'Magnitude' or 'Proportion'? 2) You have tables without heading: correct it; 3) There is discrepancies of results in the abstract and main body | 1) Proportion is better, and revision effected accordingly 2) Thank you for your concern, but. We did not get a table without heading (maybe there are tables that spanned more than one page large). 3) We have added the suggested content to the result part of the abstract line numbers 31-42 |
| 10) Discussion: 1) Is that, 'zero fruit and vegetables' or 'zero fruits or vegetables'? Be consistent in your wording; 2) Very shallow discussion [it has no implications to nutrition interventions, clinical settings, research, policy, and programs]: please include these in the discussion | 1) Thank you again for your point here. Zero fruits/vegetables is the appropriate one and revision effected accordingly 2) Thank you for pointing this out. We have revised accordingly. Kindly look through line numbers 309 to 320 ,327 to 337 and 344 to 356, and 360 to 364. |
| 11) Conclusion: 1) There is discrepancies in the main body and abstract section of the manuscript [especially, your first objective is somewhat different in both sections]: focus on your objectives, and findings while giving concluding remarks | Thank you, and we have modified it accordingly. Kindly look at line number 44 and 385 |
| 12) Ethical considerations: 1) Ethical approval is required for research projects or manuscripts that used secondary data that includes personal data [data that relates to identifiable living persons]. However, your statement says, ethical approval was not your issue. How do you comment on this? | Thank you for pointing this out. There are no names of individuals or household addresses in the data files. The geographic identifiers only go down to the regional level (where regions are typically very large geographical areas encompassing several states/ provinces). Furthermore, the data is publicly available, and all ethical procedures were done by the institutions that funded, commissioned, and managed the surveys. Permission for data access was obtained from DHS through the online request from [http://www.dhsprogram.com](http://www.dhsprogram.com/). Those points are mentioned under the ethical consideration of the manuscript. Published research articles which used DHS data without further ethical approval are available elsewhere (<https://doi.org/10.1371/journal.pone.0272849> ,  <https://journals.plos.org/plosone/article?id=10.1371/journal.pone.0283629>  ). We believe that further ethical clearance will not add any value for this work/amount to the breach of any ethical procedure. |
| 13) Abbreviations: The expanded form for 'ANC' is Antenatal Care, not Ante Natal Care: Please correct that one | We are grateful for pointing this out. We have revised it accordingly kindly look at line number 394 |
| 14) General comments: 1) The manuscript is highly affected by grammatical and punctuation errors. Therefore, it is highly recommended to consult an English Expert who will work on standard and plain English so that you manuscript will be more suitable for publication and reading; 2) There is inconsistency of using major keywords throughout the document; please be serious on this issue, and try to use same wording all the way through; 3) There is slight difference in the affiliation statements between the online submission and in the manuscript; try to make them identical | 1) We agree. Thank you for the suggestions, and the manuscript has been reviewed for major spelling and grammatical errors. 2) Thank you for pointing this out. Inconsistencies have been corrected. 3) Thank you again, and this will be corrected during the submission of the revised manuscript |

| **Reviewer #2 comments** | **Responses** |
| --- | --- |
| Summary: dig out such developing countries problem is important but showing the magnitude at local level is more significant, because the data obtained from this research finding is almost defined in the DHS report also. | Thank you! We believe that our study adds more detailed information besides the one reported in the DHS report. First of all, DHS report did not contain direct information on the magnitude of Zero fruits/vegetables consumption. Secondly DHS report did not identify possible factors that has a link with Zero fruits/Vegetables consumption. |
| 1) Background: line 63: better to rephrase it | Thank you for pointing this out. We have rephrased it. Kindly look at line number 63 to 65 |
| 2) Methodology: Line 146-149: you mentioned a contradictory idea with your justification. Amendment on your justification is required. If it is so your study doesn’t show any new findings than the previous studies you cited. Do you think wealth index is an individual level variable? Did you generate WI again or you took from the report of EMDHS? When you classify poor, middle or rich, the minimum required number??? When we say there is media exposure, better to operationalized??? | We are grateful for those insightful comments. We have modified the justification kindly look through line numbers 96 to 105  Wealth index is household level variable. The EMDHS datasets contained a variable labeled as wealth index and coded as “poorest”, “poorer”, “middle”, “richer”, and “richest”. For this study, we recoded it into three categories “poor” (includes the poorest and the poorer categories), “middle”, and “rich” (includes the richer and the richest categories).Media exposure was a composite variable obtained by combining whether there was a radio and /or TV in the respondent’s household with a value of“0” if a woman who didn't have either TV or Radio in her household and “1” if a woman has access to either of the media. We have included this operational definition in the methodology. And we have changed the term to media access. Kindly look through line number 153 to 162. |
| 3) Line 161: what is your weighting number??? | The new weighting variable(wt) was generated by dividing sampling weight (v005) by 1000000(1 million).The DHS suggests to always divide the sampling weight by 1,000,000 (1 million) <https://dhsprogram.com/publications/publication-DHSC19-DHS-Curriculum.cfm>  . |
| 4) Line165: Have checked it and violated the assumptions or based on your statistical knowledge? | We have used both our statistical knowledge as well as the ICC, and MOR values. |
| 5) Line 172-73: the ultimate use of LLR, deviance or AIC is similar. Why not you used either of one??? | Thank you for pointing out this. We chose deviance. Kindly take a look at line number 240 to 245. |
| 6) Line 193: large central region??? Better to operationalize. | We appreciate for your comment, the operational definition included. kindly look at line number 160 to 163 |
| Result, discussion   7) Line 198: Most of sample were from poor house household….. Will biased your findings. Needs management. Narration and table similar….. | We respect your concern. However, EMDHS was conducted based on a nationally representative sample that provided estimates at the national and regional levels and for urban and rural areas. And, since the objective of this study is to generate baseline data for the whole country, we did not see the benefit of doing subgroup analysis. Besides, the number of participants from poor households and rich households were almost comparable 41%, and 39.7% respectively. |
| 8) Line 206: Is the 95% confidence interval wide or narrow? Why? | The width of confidence interval depends on several factors, including the sample size, level of confidence, and the variability of the study participants. What is considered a “wide” or” narrow “confidence interval can vary depending on the judgment of different experts. We believe that the confidence interval is not too wide/too narrow. |
| 9) Line 215-table: 1-have checked chi-square assumptions? And if the difference in the geopolitical regions were significant, what did you think about the regression you did? | We respect your concern. The thing is significant difference after a chi-square test means that the null hypothesis of no association between geopolitical region and zero fruits/vegetables consumption is rejected. So, the geopolitical regions can be a potential confounder and needed to be included in the multivariable analysis. Our regression model follows this description and we believe that it was right. |
| 10) Line 228-229: Does the interpretation give a logical sense??? | Thank you for pointing this out. The interpretation is rephrased. Kindly look at line number 236-239 |
| 11) Table 2: if you obtain similar values for them, and between them, which is better for multilevel model comparison if any? | Deviance is better for multilevel modeling revised accordingly. Thank you for your insightful comment! |
| 12) Table 3: Your statistical significant variables are mostly sociodemographic and economic factors. What do you feel about these and its implications? | Dear reviewer thanks again for your concern. It would have been better to incorporate other variables to the model to decide if sociodemographic and economic factors are the sole predictors of zero fruits/vegetables consumption. However, due to the secondary nature of the data, we could not include other variables. And we mentioned this as limitation of the study. None the less it shows that our findings resonate well with existing literature socio economic status significantly influence dietary habits |
| 13) Line 281-285: Dear authors, are you sure your findings are consistent with the cited reference findings??? If it is so, what is the criteria to say consistent, inline, higher, lower, opposite etc.? | Dear reviewer, we are really grateful for pointing this out. The appropriate change has been made. Kindly look at line number 287-294 |
| 14) Line 289-291: if the participants were different, can we use them for comparison? | Thank you again for your concern. The recommended way of writing discussion is to compare the result with comparable participants. This happened because of paucity of research articles on the area. |
| 15) Line 296: most of your participants are from poor households…… it is difficult to assure a true statistical significance……… if the sample size for all categories is high, it might not be significant. Do you have any method you used for statistical significant assurance??? | Dear reviewer thanks for your point. But the number of participants from poor households and rich households were comparable 41%, and 39.7% respectively. So, we did not see the benefit of conducting stratified analysis by wealth index. |
| 16) Line 329: Your interpretation is inappropriate, the religion you mentioned does not recommend fasting for populations like your participants. Needs revision or better justification. | We think this is an excellent view. We have amended the justification for the observed difference, and also, we have cited a reference that showed the link between parental fasting and child feeding. Kindly look through line number 367 to 370 |
